# Effect of the Wii Sports Resort on Mobility and Health-Related Quality of Life in Moderate Stroke

**DOI:** 10.1155/2021/6635298

**Published:** 2021-06-28

**Authors:** Iratxe Unibaso-Markaida, Ioseba Iraurgi

**Affiliations:** Faculty of Psychology at University of Deusto, 48007 Bilbao, Basque Country, Spain

## Abstract

**Background:**

Stroke is a common cerebral circulatory disorder that has several sequelae that affect the daily life of patients as well as their quality of life and the lives of people close to them. Video games are being used in the rehabilitation process to address these sequelae and their benefits are shown on physical activity and in the cognition area. However, their effects on daily life activities and quality of life are not known. This study was aimed to test the effect of the Nintendo Wii Sports Resort on mobility and health-related quality of life among patients who have suffered a moderate stroke.

**Methods:**

A prepost design study was conducted with 30 moderately impaired stroke patients aged 65 ± 15. The study lasted eight weeks. 15 participated in the intervention group and the others belong to the control group. They were assessed in mobility (Timed Get Up and Go Test—TUG) and health-related quality of life (SF-36 Health Questionnaire). Parametric test and effect sizes were used to analyze the change in outcomes and to compare groups.

**Results:**

There were no differences at baseline between the groups. After the intervention, the intervention group had experienced a greater change according to the size of the effect on the variables concerning TUG (*d* = 1.32), physical function (*d* = 1.32), social function (*d* = 0.71), and Physical Component Summary (*d* = 0.75). On the other hand, the control group had a significant change in mental health according to effect size; however, this effect is not statistically relevant (*d* = 0.88; CI 95% = −3.74 to 5.50).

**Conclusions:**

The results on mobility and health-related quality of life indicate an improvement in both groups. However, according to the effect sizes and its confidence of interval, the intervention group achieved better results than the control group. Although more studies are needed in this area, the results are encouraging for improving mobility and health-related quality of life after stroke.

## 1. Introduction

Stroke is a very common cerebrovascular disease [1] which causes a worsening in motor functions and cognitive functions [2–4]. Various interventions can be carried out to reduce the sequelae of stroke, most of which have particularly focused on the physiotherapy as a rehabilitation method to improve mobility and gait [5–7]. However, patients have described the conventional rehabilitation as repetitive and have experienced decreased motivation as the session's progresses [8]. For this reason, the use of video games [9] and virtual reality [10] as therapy methods has been incorporated into rehabilitation in recent years, which has been welcomed by patients [9].

The Nintendo Wii is among the video games most frequently used in rehabilitation with patients who have suffered a stroke [11]. Evidence has been found of its use in recovery from mobility problems [12], gait difficulty [5, 13], and balance disorders [14, 15]. The Nintendo Wii has also been used in the cognitive rehabilitation of these patients [16, 17], and some studies have been conducted to compare the use of the Nintendo Wii with other types of video games, and the cognitive effect that video games have on patients [18]. In addition, virtual reality is being used with promising expectations in the recovery of physical functioning (balance and gait ability) in patients with stroke [10].

While the physical and cognitive aspects of this pathology have been studied, and research is relatively readily available [19–21], little literature exists regarding the quality of life after a stroke [22] using exergames. Therefore, the main objective of this study is to analyze the changes that occur in terms of mobility and health-related quality of life after engaging in physical activity using the console Nintendo Wii with the game Sports Resort in people who have suffered a moderate stroke.

## 2. Materials and Methods

### 2.1. Participants

Thirty patients who had suffered a moderate stroke (21 ischemic and 9 hemorrhagic) [16] participated voluntarily in the study after were informed and signed the consent. They were recruited in a hospital during their recovery period after being admitted for the cerebrovascular accident. The average age of the participants was 65 (SD = 15), and they were mainly men (67%). The inclusion criteria were being of legal age (+18), having been diagnosed as having suffered a moderate stroke using the Oxfordshire Community Stroke Project (OCSP) instrument, a score on the Barthel Scale of between 60 and 90, Mini-Mental (MMSE) with a cut-off of 23 or higher, having suffered a stroke at least one month ago, but not more than one year ago and those the preservation of the dominant hand in order to conduct the assessment tasks. The exclusion criteria were patients who do not follow inclusion criteria and after a stroke who are in unstable clinical condition or are suffering from complications that require active medical treatment, people with comprehension difficulties that prevent them from following verbal instructions, people with dementia or cognitive impairment, and people with uncontrolled psychiatric illnesses. The ethics committees from the hospital and university approved the study.

### 2.2. Measures

A number of sociodemographic and clinical variables were recorded on an ad hoc basis, as well as two instruments to be detailed later. All of them were used for both this study's purposes and as part of a wider assessment [17].

#### 2.2.1. The Timed “Up and Go” Test (TUG) [23]

The TUG measures the individual's mobility to see if they can walk independently. Support devices may be used by people who need them. In this, the patient needs to be seated on a chair with back and arm supports. Then, the individual is asked to stand up from the chair, walk a distance of 3 meters, turn around, and sit down again adopting the original position. The patient is asked to do this once to assess their mobility and, then, to repeat this task 3 times. The three trials are timed (seconds) and the average time is calculated. If the person has an average of less than 10 seconds, they are considered to be “independently mobile”; if their average is less than 20 seconds, they are deemed to be “mostly independent;” people with an average between 20 and 29 seconds are deemed to have “variable mobility,” and those with an average higher than 29 seconds are considered to have “reduced mobility.” The test-retest and interjudge reliability was .99 [24].

#### 2.2.2. SF-36 V2 Health Questionnaire [25, 26]

This is one of the most used questionnaires for the evaluation of the health-related quality of life in both the normal and clinical population. It consists of 36 items that assess health-related positive and negative aspects. These items cover 8 domains of mental and physical health. The domains are physical functioning, role-physical, bodily pain, general health, vitality, social functioning, role-emotional, and mental health. It also allows two score summaries to be obtained: the Physical Component Summary and the Mental Component Summary. The scores range from 0 (worse health status) to 100 (best health status). All domains have a reliability greater than .75, and even greater (“physical functioning,” “role-physical,” and “role-emotional” have an internal consistency >.90), except for the “social functioning” domain (*α* = .74) [27].

### 2.3. Procedure

A naturalistic prepost facto study was conducted during nine months. During the first week of admission to the rehabilitation area (first 4-5 days), the participants were evaluated by the medical team, and their suitability to participate in the study was assessed. Candidates were invited to participate, and their informed consent was requested. Once their consent had been provided, the participants were evaluated during the second week of admission (7th-8th day after admission) to establish the baseline regarding their mobility responses (TUG) and perception of their quality of life (SF-36). Subsequently, half of the participants (*n* = 15) were assigned to an intervention group using the Nintendo Wii video game, while the other half became part of the control group. All the participants received the standard rehabilitation protocol (physiotherapy, occupational therapy, speech therapy, ambulation with a walker and a healthcare support worker, and neuropsychological support) that was implemented in the hospital. The intervention supported by the use of the Nintendo Wii video game was carried out throughout 24 sessions over a period of eight weeks (three sessions/week for a maximum duration of 30 minutes with 10 minutes for rest) preferably individually, and in some cases in pairs (from session 20). In the ninth week after the baseline assessment, a second assessment was made (posttest measure) with the same instruments used at the beginning. After the posttest measure, the control group was invited to realize the intervention.

### 2.4. Data Analysis

The mean (M) and standard deviation (SD) were calculated for scalar variables and frequency (*n*) and percentage (%) for nominal variables. Normality that was calculated through the Shapiro-Wilk test and *U* Mann–Whitney test was carried out for baseline measures. The means were compared by using Student's *t*-test, both for the comparison between groups (*t*-test independent) and intragroup comparison (*t*-test pairs), with effect sizes being calculated (Cohen's *d*) for both types of comparisons and confidence intervals of Cohen's *d*. The omnibus test was carried out by means of an analysis of the variance of repeated measures between two groups, thus estimating the intergroup, intragroup, and interaction effects. As well, to evaluate the therapeutic change, it was used the Reliable Change Index methodology purposed by Jacobson and Truax [28], which is classified the evolution of patients in three areas such as improve, no change, or worsening [29]. The contrast of classification for each group has been assessing through Chi-Square Test.

These analyses were performed with SPSS 22 version [30]. In addition, Cohen's *d* was calculated with Lakens spreadsheet [31], and the confidence intervals were performed by Wuensch's formula [32].

## 3. Results

Table 1 presents the data for the baseline evaluations for both the intervention group (IG) and the control group (CG). Effect sizes are also shown (Cohen's *d*) for the changes and normality were calculated by the Shapiro-Wilk test. Five of eleven factors were normal (*p* > .05). Despite not following a normal distribution, this study followed the recommendation of Blanca's article [33]; where in nonnormal data, it is allowed to use parametric tests. Looking at the baseline scores, a statistically significant difference was only observed for the mental health dimension of the SF-36 (*t* = 2.31; *df* = 28; *p* = .028; *d* = .84), where the intervention group was found to have better mental health than the control group (*M*_IG_ = 69.33 vs. *M*_CG_ = 53.87). A marginal statistical difference was also identified (*t* = 1.82, *df* = 28, *p* = .079; *d* = .67) in the TUG assessment, where the control group showed better performance (*M*_CG_ = 21.93) than the intervention group (*M*_IG_ = 28.73).

Table 2 presents the postintervention data and the differences between the first and second measures. It also shows the effect sizes and their interval of confidence for both groups. Based on the effect sizes obtained by measuring the changes between the baseline and the follow-up measures for both groups, it was observed that the effect size for TUG was higher in the intervention group (*d*_IG_ = 1.32) than in the control group (*d*_CG_ = 0.93), which means that while the acceleration of change was evident in both groups, it was greater in the intervention group. A similar result was observed in the physical function (*d*_IG_ = 1.32 vs. *d*_CG_ = .96), in the social function (*d*_IG_ = .71 vs. *d*_CG_ = .53), and in the case of the Physical Component Summary (*d*_IG_ = .75 vs. *d*_CG_ = .68). In all these cases, the acceleration or the magnitude of change was higher in the intervention group than in the control group. However, within those situations where there were notable effects, an important change effect was observed for mental health in the control group (*d*_CG_ = .88), which in the case of the intervention group was moderate (*d*_IG_ = .36). Nevertheless, attending to the confidence interval of Cohen's *d*, this change is not statistically significant.

**Figure 1:**
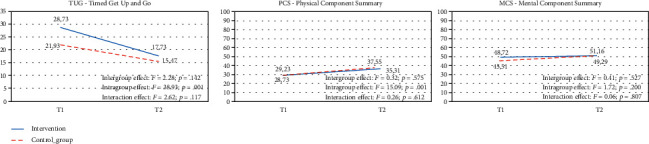
ANOVA of repeated measures by group to evaluate Timed Get Up and Go (TUG), Physical Component Summary (PCS), and Mental Component Summary (MCS).

Figure 1 presents the intergroup, intragroup, and interaction effects of the three main factors. TUG shows a statistically significant intragroup effect, but it does not present intergroup or interaction effects; also, Physical Component Summary followed the same results as TUG. However, the Mental Component Summary was not shown any significant result.

**Figure 2:**
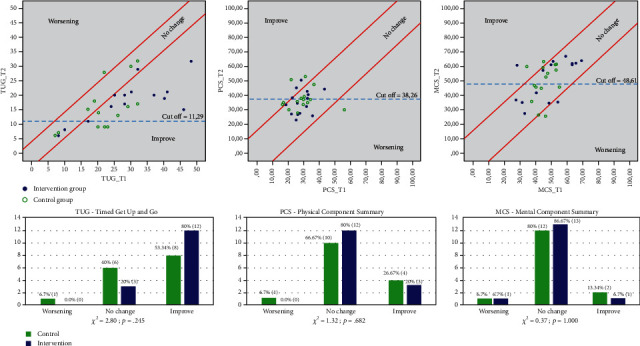
Reliable Change Index (RCI) to evaluate Timed Get Up and Go (TUG), Physical Component Summary (PCS), and Mental Component Summary (MCS).

Figure 2 presents the Reliable Change Index, which evaluates the therapeutic change in the three main factors (TUG, PCS, and MCS). The figures show three different areas depending on the punctuation of the groups. The upper graph shows the TUG where on the top of the area there were participants whose scores got worse, in the central area were those who had no change in their scores and in the lowest part were those who improved their performance. In addition, the diagram below shows these data based on numbers and percentages. It is observed that there is an improvement of 80% in the intervention group although these data are not statistically significant (*p* = .245). In addition, the rest of the upper graphs presents the data for Physical and Mental Component Summary where on the top of the area, there were participants whose scores improve, in the central area were those who had no change in their scores and in the lowest part were those who got worsening results in these factors. Both factors indicated that the main group of participants for both groups were in the middle of the area, so there was no change in their scores. In addition, these results were not statistically significant (*p* > .05).

## 4. Discussion

The main aim of this study was to analyze the changes in the mobility and health-related quality of life after doing physical activity with the Nintendo Wii Sports Resort in people who have suffered a moderate stroke.

The improvement in mobility after a stroke by applying the traditional procedures included in the rehabilitation protocols has already been proven [16, 17, 19, 21] and has also been confirmed in our findings. However, it has also been seen that the improvement effect observed in all participants was greater in the group that had used the Nintendo Wii, which leads to the conclusion that this practice can have a synergic effect with the standard rehabilitation plan. It would be necessary to clarify whether it is the additional exercise involved in using the Wii video game or the motivating effect of physical activity that caused the additional improvement noted. Some studies explored the correlation of the motivating effect with the adherence to doing exercise and the enhancement in the rehabilitation [34, 35]. These results, the improvement in mobility, are similar to other studies realised with Virtual Reality in balance and gait ability [36].

The results of this research are interesting, not so much because of the statistical comparisons performed (which have certain limitations due to the size of the samples), but because of the effect size found in some indicators that reflected an important clinical improvement. In this respect, the most obvious change occurred in mobility. Better baseline performance was seen from the control group with respect to the intervention group, as the intervention group started from a more disadvantaged position. However, after the intervention using the Nintendo Wii, the participants achieved a significant reduction in the execution time of the TUG (Δ = −11 seconds), which was greater than that obtained by the control group (Δ = −6 seconds). According to this, also, using Virtual Reality in rehabilitation improves patients' balance and gait ability [10].

A similar situation was reflected in physical function and social function, where the intervention group performed better at physical and social levels than the control group. However, the opposite result to that expected was found in that the greatest change in the SF-36 in the follow-up phase was observed in the mental health dimension, where the control group went from a score of 53.87 to a score of 75.73, that is, there was a change of approximately 22 points. In contrast, the intervention group, which started from a better initial mental health score (*M* = 69.33) achieved a postintervention score of 75.73, that is, it increased by only 6 points. Therefore, the greatest change in mental health was seen in the control group, but both groups obtained a functionality score in this dimension that was equal to the normative scores (*M* = 71.7) of their reference group [27]. This would explain that the intervention group could not progress as fast as the control group in mental health, since at the beginning of the treatment, the intervention group was close to the average found among a normative sample within their sociodemographic scope. These results were analogous to those reported in the literature [37, 38], and the same results were found with Virtual Reality [39]. In addition, using other video games and measuring those variables with an equivalent test (EQ-5D—European Quality of Life 5 Dimensions), the results were also similar [19].

Two important aspects regarding the design represent an important limitation of the study. First, the design used is a prepost facto naturalistic study in which randomization has not been used in the configuration of the groups. This fact could lead to selection bias and compromise the results. The second limitation has to do with the sample size *n* = 30, which for a population of 180 possible participants, would imply a sampling error of 16.4%; it also compromises the generalization of results. These aspects lead us to suggest prudence in the generalization of the results. However, the literature shows that similar studies have samples of similar size, due to the difficulties of including participants in clinical practice studies. We believe that the data provided along with other evidence already published may be part of a meta-analysis that would overcome the limitations of individual studies. However, although the limited sample size implies a reduction in the statistical power of the tests performed, the findings provided sufficiently noticeable effect sizes for them to be considered in terms of the relationship between the use of the Nintendo Wii in rehabilitation protocols for people who have suffered a stroke and the consequent improvement in their mobility and some aspects of their health-related quality of life.

## 5. Conclusions

The findings showed evidence that the use of the Nintendo Wii video game not only improved cognitive performance [15, 17], but that it also had effects on the improvement of mobility performance and perception of health, especially in the dimensions of physical functioning and social function [40]. More studies are needed in this area but the results are encouraging for improving mobility and health-related quality of life after stroke [16, 41].

## Figures and Tables

**Table 1:** *t-*test for independent sample before intervention.

Variables	Normality test		Intervention group		Control group		Contrast test				
	Shapiro-Wilk	*pS-W*	M	SD	M	SD	*t*	df	*p*	*d*	*pU M-W*
TUG	0.97	.832	28.73	12.31	21.93	7.53	1.83	28	.079	0.66	.106
SF36											
Physical functioning	0.77	.000	20.67	20.52	18.00	27.44	0.30	28	.765	0.11	.305
Physical role	0.50	.000	6.67	11.44	8.33	22.49	-0.26	28	.800	0.09	.653
Bodily pain	0.89	.006	57.80	30.39	60.93	39.59	-0.24	28	.810	0.09	.744
General health	0.95	.232	59.67	13.69	60.17	21.52	-0.76	23.73	.940	0.03	.870
Vitality	0.97	.628	58.33	25.19	46.67	19.24	1.42	28	.165	0.52	.285
Social function	0.78	.000	35.00	39.58	30.83	38.92	0.29	28	.773	0.11	.595
Emotional role	0.61	.000	68.89	44.48	84.44	33.01	-1.09	28	.286	0.40	.345
Mental health	0.97	.664	69.33	17.99	53.87	18.63	2.31	28	.028	0.84	.089
Physical component summary	0.90	.014	28.73	6.23	29.23	9.52	-0.17	28	.864	0.06	.935
Mental component summary	0.98	.850	48.72	14.11	45.51	6.26	0.80	19.30	.431	0.29	.412

TUG: Timed Get Up and Go Test; *p* S-W: significant level of normality test of Shapiro-Wilk; M: mean; SD: standard deviation; *t*: *t*-Student; df: degree of freedom; *p*: *p* value; *d*: Cohen's *d*; *p U M-W*: significant level of normality test of *U* Mann–Whitney.

**Table 2:** Outcomes changes between prepost intervention and effect sizes for both groups.

Variables	Intervention group						Control group					
	After		Change T2-T1				After		Change T2-T1			
	M	SD	M	SD	*d*	CI	M	SD	M	SD	*d*	CI
Timed Get Up and Go (TUG)	17.73	7.09	-11.00	8.35	1.32	0.71 to 1.92	15.47	8.40	-6.46	6.91	0.93	-3.91 to 5.77
SF36												
Physical functioning	52.33	21.12	31.66	23.89	1.32	0.56 to 2.08	59.00	23.24	41.00	42.81	0.96	-4.09 to 6.01
Physical role	18.33	32.00	11.66	31.15	0.37	-0.26 to 1.00	21.67	32.55	13.34	44.18	0.30	-1.45 to 2.05
Bodily pain	51.53	31.85	-6.27	26.47	0.24	-0.19 to 0.68	70.80	35.70	9.87	32.05	0.30	-1.31 to 1.91
General health	67.27	25.54	7.60	23.28	0.33	-0.22 to 0.88	64.67	13.66	4.50	22.32	0.20	-1.01 to 1.41
Vitality	63.00	26.71	4.67	23.93	0.19	-0.28 to 0.66	58.67	19.59	12.00	24.40	0.49	-2.13 to 3.11
Social function	65.00	33.47	30.00	41.94	0.71	0.07 to 1.34	50.83	38.81	20.00	37.44	0.53	-2.26 to 3.32
Emotional role	68.89	46.23	0.00	43.62	0.00	-0.49 to 0.49	80.00	41.40	-4.44	50.17	0.09	-0.73 to 0.91
Mental health	75.73	24.54	6.40	17.94	0.36	-0.06 to 0.78	75.73	17.27	21.86	24.84	0.88	-3.74 to 5.50
Physical Component Summary	35.31	8.34	6.58	8.79	0.75	0.09 to 1.41	37.55	7.48	8.32	12.23	0.68	-2.92 to 4.28
Mental Component Summary	51.16	14.20	2.44	12.48	0.19	-0.26 to 0.64	49.29	12.38	3.78	12.25	0.31	-1.41 to 2.03

M: mean; SD: standard or typical deviation; *d*: Cohen's d; CI: confidence interval of Cohen's *d*.

## Data Availability

The data used to support the findings of this study are available from the corresponding author upon request.
